# Does body mass index influence improvement in patient reported outcomes following total knee arthroplasty? A retrospective analysis of 3918 cases

**DOI:** 10.1186/s43019-023-00195-1

**Published:** 2023-07-26

**Authors:** Kyle W. Lawrence, Walter Sobba, Vinaya Rajahraman, Ran Schwarzkopf, Joshua C. Rozell

**Affiliations:** Department of Orthopedic Surgery, NYU Langone Health, 301 East 17th Street, 15th Fl Suite 1518, New York, NY 10003 USA

**Keywords:** Knee, Obesity, Total knee arthroplasty, Body mass index, Patient reported outcomes

## Abstract

**Purpose:**

The study aimed to determine whether body mass index (BMI) classification for patients undergoing total knee arthroplasty (TKA) is associated with differences in mean patient reported outcome measure (PROM) score improvements across multiple domains—including pain, functional status, mental health, and global physical health. We hypothesized that patients with larger BMIs would have worse preoperative and postoperative PROM scores, though improvements in scores would be comparable between groups.

**Materials and methods:**

Patients undergoing primary TKA from 2018 to 2021 were retrospectively reviewed and stratified into four groups: Normal Weight; 18.5–25 kg/m^2^, Overweight; 25.01–30 kg/m^2^, Obese; 30.01–40 kg/m^2^, and Morbidly Obese > 40 kg/m^2^. Preoperative, postoperative, and pre/post-changes (Δ) in knee injury and osteoarthritis, joint replacement (KOOS, JR) and Patient-Reported Outcome Measurement Information System (PROMIS) measures of pain intensity, pain interference, physical function, mobility, mental health, and physical health were compared. Multivariate linear regression was used to assess for confounding comorbid conditions.

**Results:**

In univariate analysis, patients with larger BMIs had worse scores for KOOS, JR and all PROMIS metrics preoperatively. Postoperatively, scores for KOOS, JR and PROMIS pain interference, mobility, and physical health were statistically worse in higher BMI groups, though differences were not clinically significant. Morbidly obese patients achieved greater pre/post-Δ improvements in KOOS, JR and global physical health scores. Multivariate regression analysis showed high BMI was independently associated with greater pre/post-Δ improvements in KOOS, JR and global health scores.

**Conclusion:**

Obese patients report worse preoperative scores for function and health, but greater pre/post-Δ improvements in KOOS, JR and physical health scores following TKA. Quality of life benefits of TKA in obese patients should be a factor when assessing surgical candidacy.

## Introduction

Obesity represents a significant modifiable risk factor for poor health outcomes and has continued to rise in prevalence in the United States and worldwide [1, 2]. The association between obesity and the development of knee osteoarthritis (OA) is well established, and the lifetime risk for symptomatic OA is increased by 30.3% in obese individuals when compared to those with a normal body mass index (BMI) [3–5]. Consequently, a growing number of patients indicated for total knee arthroplasty (TKA) are overweight or obese [6, 7], and the projected rising demand for this procedure reflects the growing rate of obesity within the population [8, 9].

The association between obesity and poor clinical outcomes following TKA has become an important area of research, as obese patients demonstrate higher rates of 30-day complications—such as infection, wound dehiscence, and venous thromboembolism—as well as in-hospital mortality [10–14]. As such, some institutions have suggested adopting BMI cutoffs when identifying TKA candidates preoperatively, despite concurrent evidence that other comorbid conditions such as malnutrition, diabetes, liver disease, and coagulopathy may confer greater operative risk than obesity alone [15–17]. In doing so, surgeons may restrict obese patients from a surgical intervention who would otherwise experience vast improvements in pain and function with an uncomplicated postoperative recovery [18].

Recent interest in quality-of-life improvement for obese patients undergoing TKA has called into question the restrictive risk factor profile for these patients. Prior analyses of patient-reported outcome measures (PROMs) in obese and non-obese patients have demonstrated that obese patients have worse pain and function scores preoperatively [19, 20]. However, investigations of absolute postoperative PROM scores and score improvements for obese and non-obese patients undergoing TKA have generated heterogeneous results [21–24]. For example, Collins et al. followed a group of 633 TKA patients stratified by BMI, finding that obese patients had greater improvements in pain and function from baseline to 3 months, and achieved comparable absolute function and pain relief to non-obese patients at 24 months [21]. In contrast, Mohammad et al. analyzed a larger group of 4750 TKA recipients and found obesity to be independently associated with worse postoperative pain, though they did not analyze other PROM domains [22]. Li et al. analyzed 2964 TKA patients from a national database and found that obese patients had comparable improvements in functionality to non-obese patients, despite achieving lower absolute functional status at 6 months postoperatively [23]. Though functional status remained lower postoperatively for obese patients in their study, pain relief was greater than in their non-obese counterparts. In contrast, Chen et al. reviewed two validated measures of functionality in 7733 TKA recipients, finding that obese patients had greater score improvements with surgery than did non-obese patients [24]. Thus, no consensus yet exists regarding how BMI may impact PROMs following TKA.

The study aimed to determine whether BMI classification for patients undergoing TKA is associated with differences in mean PROM score improvements across multiple domains—including pain, functional status, mental health, and global physical health. We hypothesized that patients with larger BMIs would have worse preoperative and postoperative PROM scores, though improvements in scores would be comparable between groups.

## Methods

### Study design

Our institution began collecting perioperative PROM surveys on all TKA patients in 2018. Therefore, we retrospectively reviewed all patients undergoing primary, elective TKA at our academic center between 2018 and 2021. Patients were excluded from analysis if they underwent simultaneous bilateral, revision, unicondylar, or non-elective TKA. PROMs were extracted from the electronic medical record and included the knee injury and osteoarthritis, joint replacement (KOOS, JR) score as well as Patient-Reported Outcome Measurement Information System (PROMIS) measures of pain intensity, pain interference, physical function, mobility, global mental health, and global physical health. Institutional review board (IRB) approval was obtained prior to the start of the study.

### Patient demographics and stratification

Patient baseline characteristics (BMI, age, sex, self-identified race, and smoking status) and comorbidities were retrospectively extracted from the electronic medical record. BMI was used to stratify patients into four groups (Normal Weight: BMI 18.5–25 kg/m^2^, Overweight: BMI 25.01–30 kg/m^2^, Obese: BMI 30.01–40 kg/m^2^, and Morbidly Obese: BMI > 40 kg/m^2^) based on the World Health Organization (WHO) guidelines [25]. At our institution, no current BMI cutoff exists, and obese patients who are medically cleared for surgery can undergo TKA. Only eight patients with a BMI < 18.5 kg/m^2^ underwent TKA at our institution during the study period and had completed PROM surveys, and thus these patients were excluded from analysis. Baseline demographics were compared between groups. A total of 11,151 knee arthroplasty procedures occurred during the study period. After removing simultaneous bilateral, revision, unicondylar, and non-elective procedure indications, 9343 primary TKAs remained. Of those, 3305 patients undergoing 3918 TKA procedures had preoperative and postoperative PROMs and were included in our analysis: 409 (10.4%) in the normal weight group, 1170 (29.9%) in the overweight group, 1875 (47.9%) in the obese group, and 464 (11.8%) in the morbidly obese group (see Table 1). Average follow-up time was 1.1 ± 1.3 years. Average BMIs were 22.7 ± 1.7, 27.7 ± 1.4, 34.4 ± 2.8, and 43.8 ± 4.5 kg/m^2^ in the normal weight, overweight, obese, and morbidly obese groups, respectively. All groups were predominantly female, though the normal weight and morbidly obese groups had the largest female preponderance (*p* < 0.001). Average age at surgery decreased as obesity class increased (*p* < 0.001). Obese and morbidly obese patients were more predominantly non-white (*p* < 0.001). The following comorbid diagnoses displayed a significant difference among BMI groups and were included with patient demographics as independent variables in the multiple linear regression models: myocardial infarction (*p* = 0.009), cerebrovascular disease (*p* = 0.008), chronic pulmonary disease (*p* = 0.003), liver disease (*p* = 0.041), diabetes without chronic complications (*p* < 0.001), diabetes with chronic complications (*p* = 0.007), leukemia/lymphoma (*p* = 0.002), and metastatic solid tumor (*p* = 0.013). Diabetes, liver disease, and pulmonary disease were higher in the obese groups. Full comparison of comorbidities by BMI class can be found in Table 2.

**Table 1:** Patient demographics by BMI classification

	18.5–25 kg/m^2^	25.01–30 kg/m^2^	30.01–40 kg/m^2^	> 40 kg/m^2^	*P*-value
	*n* = 409	*n* = 1170	*n* = 1875	*n* = 464	
Age (years)	69.8 [27: 89]	68.5 [28: 90]	65.6 [28: 92]	61.7 [34: 85]	**< 0.001**
BMI (kg/m^2^)	22.7 ± 1.69	27.7 ± 1.42	34.4 ± 2.77	43.8 ± 4.51	**< 0.001**
Sex					
Female	308 (75.3%)	653 (55.8%)	1218 (65.0%)	343 (73.9%)	**< 0.001**
Male	101 (24.7%)	517 (44.2%)	657 (35.0%)	121 (26.1%)	
Race					
White	286 (69.9%)	744 (63.6%)	1068 (57.0%)	278 (59.9%)	**< 0.001**
African American (Black)	34 (8.3%)	153 (13.1%)	399 (21.3%)	109 (23.5%)	
Asian/Pacific Islander	33 (8.1%)	82 (7.0%)	55 (2.9%)	11 (2.4%)	
Other	56 (13.7%)	191 (16.3%)	353 (18.8%)	66 (14.2%)	
Smoking status					
Current	14 (3.4%)	61 (5.2%)	105 (5.6%)	21 (4.5%)	0.443
Former	156 (38.1%)	464 (39.7%)	720 (38.4%)	194 (41.8%)	
Never	239 (58.4%)	643 (55.0%)	1045 (55.7%)	248 (53.4%)	

Bold indicates statistical significance

*BMI* body mass index

**Table 2:** Baseline comorbidity incidence compared between groups for use in multiple linear regression analysis

	18.5–25 kg/m^2^	25.01–30 kg/m^2^	30.01–40 kg/m^2^	> 40 kg/m^2^	*P*-value
Myocardial Infarction	5 (1.4%)	37 (3.7%)	28 (1.8%)	12 (3.3%)	**0.009**
Congestive Heart Failure	8 (2.2%)	30 (3%)	57 (3.6%)	16 (4.4%)	0.317
Peripheral Vascular Disease	17 (4.7%)	80 (7.9%)	116 (7.4%)	31 (8.5%)	0.152
Cerebrovascular Disease	35 (9.6%)	101 (10%)	110 (7%)	20 (5.5%)	**0.008**
Dementia	2 (0.5%)	7 (0.7%)	6 (0.4%)	0 (0%)	0.372
Chronic Pulmonary Disease	60 (16.5%)	179 (17.7%)	277 (17.7%)	94 (25.7%)	**0.003**
Rheumatic Disease	30 (8.2%)	74 (7.3%)	109 (7%)	19 (5.2%)	0.407
Peptic Ulcer Disease	11 (3%)	34 (3.4%)	50 (3.2%)	8 (2.2%)	0.755
Mild Liver Disease	15 (4.1%)	42 (4.2%)	84 (5.4%)	29 (7.9%)	**0.041**
Severe Liver Disease	0 (0%)	1 (0.1%)	4 (0.3%)	1 (0.3%)	0.737
DM without Chronic Complications	37 (10.2%)	185 (18.3%)	353 (22.6%)	85 (23.2%)	**< 0.001**
DM with Chronic Complications	3 (0.8%)	31 (3.1%)	41 (2.6%)	18 (4.9%)	**0.007**
Hemiplegia/Paraplegia	0 (0%)	5 (0.5%)	1 (0.1%)	2 (0.5%)	0.057
Renal Disease	6 (1.6%)	47 (4.6%)	68 (4.4%)	16 (4.4%)	0.056
Malignancy: Leukemia/Lymphoma	57 (15.7%)	126 (12.5%)	150 (9.6%)	32 (8.7%)	**0.002**
Malignancy: Solid Tumor	20 (5.5%)	39 (3.9%)	38 (2.4%)	9 (2.5%)	**0.013**
HIV/AIDS	3 (0.8%)	16 (1.6%)	14 (0.9%)	2 (0.5%)	0.307

Bold indicates statistical significance

*DM* diabetes mellitus

In addition, baseline demographics and all-cause revision rates at latest follow-up were compared between patients with PROM scores included in our study (+ PROM) and patients undergoing TKA during the study period who were excluded due to incomplete PROMs (− PROM). A total of 5162 patients (5,425 TKAs) were excluded due to incomplete PROMs. Patients with PROMs were less predominantly female (+ PROM: 64.5% vs − PROM: 68.7%, *p* < 0.001) and non-White (+ PROM: 39.3% vs − PROM: 45.7%, *p* < 0.001) compared to those without PROMs. Those with PROMs were slightly younger (+ PROM: 65.7 ± 9.6 vs − PROM: 66.9 ± 9.4, *p* < 0.001) and had slightly lower average BMIs (+ PROM: 32.0 ± 6.3 vs − PROM: 32.6 ± 6.2, *p* < 0.001), but both differences were not clinically significant. Charlson Comorbidity Index scores were comparable (+ PROM: 3.2 ± 2.3 vs − PROM: 3.3 ± 2.3, *p* = 0.261). All-cause revisions at latest follow-up were greater for patients with PROMs (+ PROM: 2.9% vs − PROM: 1.6%, *p* < 0.001).

### Patient reported outcome measures

Preoperative PROMs were analyzed from patients’ latest office visit prior to surgery. Postoperative PROMs were recorded at patients’ postoperative follow-up appointments. Patients who did not have both a preoperative and postoperative score reported for at least one outcome during the study period were excluded from analysis. Given that the aim of this study was to assess overall differences in PROM score improvements across BMI classes, rather than absolute score changes within a specific postoperative time frame, the latest score completed during the study period was used for patients with multiple postoperative scores reported. Pre/Post- PROM score change (Pre/Post-Δ) was calculated by subtracting the preoperative score from the postoperative score. Average preoperative, postoperative, and Pre/Post-Δ scores were compared between all groups.

### Data analysis

Categorical variables were compared using chi squared analysis, and continuous variables were compared using analysis of variance (ANOVA). Due to the association between obesity and other comorbidities, robust multiple linear regression analyses were performed to assess for confounding effects of demographics and patient comorbidities on PROMs. Fisher’s exact tests were used to compare the prevalence of comorbid diagnoses between BMI groups. Baseline demographics and comorbid diagnoses that displayed a significant difference among BMI groups were included as independent variables in the robust multiple linear regression models. Dependent variables for regression models were postoperative and pre-/post-Δ scores for KOOS, JR and PROMIS global physical health. All robust multiple regression models fulfilled all conditions for multicollinearity, multivariate normality, and linear relation to outcome variables. Categorical variables are represented as count (percentage). Age is presented as mean [range]. BMI ranges were set during group assignment and are thus represented as mean ± standard deviation. KOOS, JR raw scores were converted to interval scores [range 0–100] [26], and PROMIS raw scores were converted to T-scores normally distributed about a mean of 50 and standard deviation of 10 in a validated reference population [27]. PROM scores were presented as mean ± standard deviation. Date ranges are represented as mean ± standard deviation. Regression results are presented as estimate effect [95% confidence interval (CI)]. Significance was determined at *p* < 0.05. Data analysis was conducted using the software R (Version 4.0.2, The R Foundation, 2022) in conjunction with RStudio (Version 1.3.959, RStudio Team, 2022).

## Results

### Univariate analyses

Preoperative KOOS, JR scores were significantly lower in higher BMI groups (*p* < 0.001) (Table 3). Postoperative KOOS, JR scores were slightly higher in the normal weight group than in the overweight, obese, and severely obese groups (*p* = 0.047). There was a greater pre/post-Δ improvement in KOOS, JR as obesity class increased: 14.6 ± 17.7, 15.9 ± 17.1, 18.3 ± 17.8, and 21.4 ± 19.6 0 in the normal weight, overweight, obese, and morbidly obese groups, respectively (*p* < 0.001) (see Table 3).

**Table 3:** Preoperative, postoperative, and pre/post-change (Δ) KOOS, JR scores stratified by BMI classification

BMI	18.5–25 kg/m^2^	25.01–30 kg/m^2^	30.01–40 kg/m^2^	> 40 kg/m^2^	*P*-value
KOOS, JR	*n* = 206	*n* = 569	*n* = 925	*n* = 219	
Preoperative	51.7 ± 14.4	48.8 ± 14.0	45.0 ± 15.1	44.4 ± 15.0	**< 0.001**
Postoperative	66.4 ± 15.2	64.7 ± 15.4	63.3 ± 15.0	64.2 ± 16.8	**0.047**
Δ	14.6 ± 17.7	15.9 ± 17.1	18.3 ± 17.8	21.4 ± 19.6	**< 0.001**

Bold indicates statistical significance

*BMI* body mass index, *KOOS, JR* knee injury and osteoarthritis, joint replacement

All preoperative PROMIS metrics were worse for obese and morbidly obese patients (see Table 4). Postoperatively, only measures of pain interference (*p* < 0.002), mobility (*p* < 0.001), and physical health (*p* < 0.001) were statistically worse among obese patients, though absolute score differences were not large. A difference in pre/post-Δ scores was only observed for physical health, and was greater for higher obesity classes (normal weight: 2.6 ± 7.9 vs overweight: 3.5 ± 7.4 vs obese: 3.8 ± 7.5 vs morbidly obese: 5.2 ± 7.3, *p* = 0.003).

**Table 4:** Preoperative, postoperative, and pre/post-change (Δ) PROMIS scores stratified by BMI classification

BMI	18.5–25 kg/m^2^	25.01–30 kg/m^2^	30.01–40 kg/m^2^	> 40 kg/m^2^	*P*-value
PROMIS—pain intensity	*n* = 231	*n* = 621	*n* = 986	*n* = 244	
Preoperative	52.4 ± 7.2	53.1 ± 7.2	54.6 ± 7.6	55.7 ± 7.3	**< 0.001**
Postoperative	46.7 ± 8.7	47.0 ± 8.4	47.9 ± 8.9	47.8 ± 8.7	0.061
Δ	− 5.7 ± 9.5	− 6.1 ± 9.3	− 6.7 ± 10.2	− 7.9 ± 9.9	0.059
PROMIS—pain interference	*n* = 244	*n* = 655	*n* = 1048	*n* = 256	
Preoperative	62.3 ± 6.9	63.3 ± 6.9	65.2 ± 7.1	65.7 ± 6.4	**< 0.001**
Postoperative	56.8 ± 8.9	57.3 ± 8.6	58.6 ± 9.0	58.8 ± 9.3	**0.002**
Δ	− 5.5 ± 9.5	− 6.0 ± 8.9	− 6.6 ± 9.4	− 6.9 ± 8.9	0.220
PROMIS—physical function	*n* = 69	*n* = 207	*n* = 311	*n* = 77	
Preoperative	37.9 ± 7.6	36.3 ± 7.1	35.6 ± 6.7	34.5 ± 6.8	**0.017**
Postoperative	40.6 ± 8.5	40.1 ± 7.7	39.9 ± 8.1	39.1 ± 7.5	0.697
Δ	2.7 ± 8.7	3.7 ± 7.5	4.3 ± 8.0	4.6 ± 7.4	0.378
PROMIS—mobility	*n* = 149	*n* = 381	*n* = 611	*n* = 146	
Preoperative	36.6 ± 4.9	36.9 ± 4.7	35.3 ± 4.8	34.6 ± 4.1	**< 0.001**
Postoperative	39.8 ± 6.7	40.7 ± 6.3	39.0 ± 6.1	38.3 ± 5.6	**< 0.001**
Δ	3.2 ± 6.3	3.8 ± 6.2	3.7 ± 6.1	3.7 ± 6.2	0.784
PROMIS—mental health	*n* = 179	*n* = 506	*n* = 777	*n* = 206	
Preoperative	49.2 ± 9.2	49.5 ± 8.6	48.1 ± 9.2	46.8 ± 9.7	**0.002**
Postoperative	49.7 ± 9.1	49.8 ± 8.7	49.3 ± 9.2	48.3 ± 9.6	0.247
Δ	0.49 ± 7.0	0.36 ± 7.3	1.2 ± 7.6	1.5 ± 7.6	0.122
PROMIS—physical health	*n* = 191	*n* = 519	*n* = 830	*n* = 212	
Preoperative	42.3 ± 7.5	41.0 ± 6.7	39.1 ± 6.9	37.1 ± 6.8	**< 0.001**
Postoperative	44.9 ± 8.2	44.6 ± 7.4	43.0 ± 7.8	42.3 ± 7.5	**< 0.001**
Δ	2.6 ± 7.9	3.5 ± 7.4	3.8 ± 7.5	5.2 ± 7.3	**0.003**

Bold indicates statistical significance

*BMI* body mass index, *PROMIS* Patient-Reported Outcome Measurement Information System

### Multivariate regression analyses

The postoperative and pre/post-Δ scores for KOOS, JR (see Table 5) and PROMIS global physical health (see Table 6) were chosen as dependent variables for regression analyses, as these outcomes were found to be statistically different between groups and represent patients’ perception of surgical success in multiple broad domains including pain, function, and overall perceived health.

**Table 5:** Multiple linear regression analysis of patient comorbidities and demographics impacting postoperative and pre/post-change (Δ) KOOS, JR scores

Baseline variable	Postoperative KOOS, JR estimate effect		Δ KOOS, JR estimate effect	
	Estimate [95% CI]	*P*-value	Estimate [95% CI]	*P*-value
Age (years)	0.2 [0.1: 0.2]	**< 0.001**	− 0.1 [− 0.2: 0.0]	0.146
Female	− 0.6 [− 1.9: 0.8]	0.420	2.8 [1.0: 4.7]	**0.003**
BMI 18.5–25 kg/m^2^ (reference)	1.0		1.0	
BMI 25.01–30 kg/m^2^	− 2.2 [− 4.4: − 0.1]	**0.045**	0.8 [− 2.2: 3.8]	0.621
BMI 30.01–40 kg/m^2^	− 2.3 [− 4.: − 0.2]	**0.034**	3.6 [0.8: 6.5]	**0.014**
BMI > 40 kg/m^2^	− 1.9 [− 4.7: 0.8]	0.163	8.0 [4.3: 11.7]	**< 0.001**
White (reference)	1.0		1.0	
African-American (Black)	− 4.9 [− 6.7: − 3.0]	**< 0.001**	− 1.3 [− 3.8: 1.2]	0.323
Asian	− 0.4 [− 3.4: 2.7]	0.803	2.2 [− 2.1: 6.4]	0.326
Other Race	− 3.2 [− 5.0: − 1.4]	**< 0.001**	0.8 [− 1.7: 3.2]	0.537
Myocardial Infarction	0.9 [− 3.4: 5.2]	0.683	0.3 [− 5.7: 6.3]	0.919
Cerebrovascular disease	− 2.9 [− 5.3: − 0.5]	**0.018**	− 2.1 [− 5.5: 1.2]	0.209
Chronic Pulmonary Disease	− 2.0 [− 3.7: − 0.3]	**0.024**	2.5 [0.1: 4.8]	**0.045**
Mild Liver Disease	− 0.1 [− 3.1: 2.9]	0.950	1.9 [− 2.4: 6.2]	0.388
DM without chronic complications	− 1.4 [− 3.1: 0.4]	0.128	0.0 [− 2.3: 2.3]	0.996
DM with chronic complications	− 3.9 [− 7.8: 0.1]	0.053	− 4.5 [− 10.0: 1.0]	0.110
Malignancy lymphoma leukemia	− 1.7 [− 3.7: 0.4]	0.107	− 2.5 [− 5.3: 0.2]	0.073
Metastatic solid tumor	1.8 [− 1.5: 5.1]	0.293	2.9 [− 1.7: 7.4]	0.210

Bold indicates statistical significance

*BMI* body mass index, *KOOS,* *JR* knee injury and osteoarthritis, joint replacement, *CI* confidence interval, *DM* diabetes mellitus

**Table 6:** Multiple linear regression analysis of patient comorbidities and demographics impacting postoperative and pre/post-change (Δ) PROMIS-PH scores

Baseline variable	Postoperative PROMIS GH estimate effect		Δ PROMIS GH estimate effect	
	Estimate [95% CI]	*P*-value	Estimate [95% CI]	*P*-value
Age (years)	0.1 [0.01: 0.1]	**0.009**	0.01 [− 0.03: 0.05]	0.562
Female	− 1.8 [− 2.5: −1.1]	**< 0.001**	0.7 [− 0.03: 1.5]	0.061
BMI 18.5–25 kg/m^2^ (reference)	1.0		1.0	
BMI 25.01–30 kg/m^2^	− 0.6 [− 1.7: 0.5]	0.293	0.6 [− 0.6: 1.8]	0.341
BMI 30.01–40 kg/m^2^	− 1.5 [− 2.5: − 0.5]	**0.005**	1.6 [0.4: 2.7]	**0.007**
BMI > 40 kg/m^2^	− 2.1 [− 3.4: − 0.8]	**0.002**	3.0 [1.6: 4.4]	**< 0.001**
White (reference)	1.0		1.0	
African-American (Black)	− 1.7 [− 2.6: − 0.8]	**< 0.001**	− 0.1 [− 1.1: 0.8]	0.800
Asian	− 2.4 [− 3.9: − 0.8]	**0.003**	1.0 [− 0.6: 2.6]	0.211
Other Race	− 3.0 [− 3.9: − 2.1]	**< 0.001**	1.2 [0.2: 2.2]	**0.018**
Myocardial Infarction	− 1.6 [− 3.8: 0.5]	0.129	− 0.3 [− 2.6: 2.1]	0.829
Cerebrovascular disease	− 0.4 [− 1.5: 0.8]	0.542	− 0.4 [− 1.7: 0.9]	0.522
Chronic Pulmonary Disease	− 1.0 [− 1.8: − 0.2]	**0.014**	− 0.4 [− 1.3: 0.5]	0.375
Mild Liver Disease	− 0.7 [− 2.2: 0.7]	0.328	− 0.3 [− 1.9: 1.2]	0.682
DM without chronic complications	− 1.6 [− 2.4: − 0.7]	**< 0.001**	− 0.1 [− 1.0: 0.9]	0.887
DM with chronic complications	− 1.9 [− 3.7: 0.0]	**0.049**	− 0.8 [− 2.9: 1.3]	0.464
Malignancy lymphoma leukemia	0.2 [− 0.8: 1.2]	0.683	0.1 [− 1.0: 1.2]	0.894
Metastatic solid tumor	− 0.3 [− 2.0: 1.3]	0.698	− 1.1 [− 2.9: 0.6]	0.211

Bold indicates statistical significance

*BMI* body mass index, *PROMIS* Patient-Reported Outcome Measurement Information System, *Global PH* Global Physical Health, *DM* diabetes mellitus, *CI* confidence interval

Regression analysis found that being overweight (*p* = 0.045) or obese (*p* = 0.034) was associated with modestly lower postoperative KOOS, JR scores. African-American (Black) or other non-White race was associated with lower postoperative KOOS, JR scores. Chronic pulmonary disease (*p* = 0.024) and cerebrovascular disease (*p* = 0.018) were associated with modestly decreased postoperative KOOS, JR scores. After adjusting for demographic factors and comorbidities, being obese and severely obese was independently associated with the greatest increase in pre-/post-∆ scores for KOOS, JR (obese: 3.6, 95%CI 0.8–6.5, *p* = 0.014; severely obese: 8.0, 95%CI 4.3–11.7, *p* < 0.001). Female sex (*p* = 0.003) and chronic pulmonary disease (*p* = 0.045) were also associated with greater improvements in KOOS, JR scores. No comorbid diagnoses were associated with inferior improvement in KOOS, JR scores (see Table 5).

Being obese (*p* = 0.005) and morbidly obese (*p* = 0.002) was associated with lower postoperative PROMIS global health scores. African-American (Black) or other non-White race was associated with lower postoperative PROMIS global health scores. Chronic pulmonary disease (*p* = 0.014) as well as diabetes with (*p* < 0.001) and without (*p* = 0.049) chronic complications were associated with modestly decreased PROMIS physical health scores. After adjusting for demographic factors and comorbidities, being obese and severely obese was independently associated with the greatest increase in pre-/post-∆ scores for PROMIS global physical health (obese: 1.6, 95%CI 0.4–2.7, *p* = 0.007; severely obese: 3.0, 95%CI 1.6 to 4.4, *p* < 0.001). No comorbid diagnoses were associated with inferior improvement in PROMIS global physical health scores (see Table 6).

## Discussion

This study compared PROM scores following TKA for patients based on BMI classification and reports the following key findings: (1) obese patients report lower preoperative and postoperative scores for most measures of functional status and health, (2) pre/post-Δ improvements in PROMIS measures of pain, mobility, function, and mental health are comparable regardless of BMI class and (3) elevated BMI is independently associated with greater improvements in KOOS, JR and PROMIS physical health scores following TKA. Importantly, the ability to optimize all other modifiable risk factors in obese patients may encourage surgeons to be less hesitant to operate on these patients for fear of an unfavorable postoperative risk profile.

Current literature on the impact of BMI on PROM improvement following TKA has yielded heterogeneous results [21–23], though accurately assessing quality-of-life outcomes based on BMI is critical to adequately counsel obese patients regarding the risks and benefits of surgical intervention. The lack of consensus regarding the influence of BMI on PROMs following TKA in the current literature may be attributable to a variety of factors, including variations in obesity classification methods, PROM measures utilized, and health status of study groups across BMI classifications [21]. Additionally, limited sample sizes due to loss to follow-up and survey non-compliance make large-scale analyses of PROMs in multiple domains challenging. Our study addresses several of the limitations associated with prior literature on this topic. We analyzed a robust sample size of patients reporting across multiple domains of pain, functional status, and overall health following an institution-wide effort to collect multiple PROM surveys for all TKA patients. Further, we adjusted for comorbid diagnoses associated with BMI to demonstrate the independent effect of obesity on PROM improvement. Finally, we compared our study population to patients who did not complete PROM scores during the study period, thus providing more insight into how these results may be generalizable to a broader population.

With regard to pain, our study showed that measures of pain intensity and pain interference were worse preoperatively for obese patients, though pre/post-Δ improvements in pain scores were comparable regardless of obesity classification. Similarly, functional status as measured by PROMIS physical function and PROMIS mobility was inferior for obese patients preoperatively, though pre/post-Δ improvements were comparable between groups. This emphasizes that despite potential biases in patient selection, obese patients can be motivated to improve their postoperative quality of life. For both PROMIS pain interference and PROMIS mobility, obese patients reported statistically worse postoperative scores, though these differences were likely not clinically significant [27, 28]. KOOS, JR scores, which evaluate knee function as well as pain, were also worse preoperatively for obese patients. Though postoperative KOOS, JR scores were slightly lower in the obese groups, these differences were likely also not clinically meaningful [29]. On average however, all BMI groups achieved clinically meaningful improvements in KOOS, JR scores after surgery, with obese groups demonstrating a significantly greater pre/post-Δ improvement in scores postoperatively [29]. This reflects an important finding of our current study; clinically meaningful differences in functional scores can be achieved regardless of BMI class, and obese patients achieve relatively greater improvements than non-obese patients. Similarly, pre/post-Δ improvements in PROMIS global physical health were greater for obese patients than non-obese patients, though all groups achieved clinically significant improvements postoperatively [30]. With regards to mental health scores, no clinically significant differences were observed between groups. Prior analyses have demonstrated that TKA improves measures of global physical and mental health, however the influence of obesity on these broad metrics remains undefined [30, 31]. We demonstrate that BMI class does not influence changes in mental health scores, though elevated BMI is associated with greater improvements in physical health scores.

After controlling for comorbidities, regression analyses found that elevated BMI was independently associated with greater pre/post-Δ improvements in KOOS, JR and PROMIS global physical health scores, though the degree of associated improvement was slight. As discussed, obese patients reported worse preoperative and postoperative KOOS, JR and PROMIS global physical health scores, though the differences in postoperative scores were likely not clinically significant. In conjunction, these findings suggest that comparable postoperative PROMs are achieved regardless of BMI class, and thus obese patients likely achieve greater score increases due to a higher preoperative potential for improvement. No comorbid conditions were independently associated with worse score improvements for KOOS, JR or PROMIS physical health metrics. Though several additional comorbidities—including myocardial infarction, diabetes, and cerebrovascular disease—were more prevalent in obese groups, these diagnoses did not influence PROM score improvement for either KOOS, JR or PROMIS global physical health. Notably however, it was observed that several comorbidities were more prevalent in obese groups, underscoring the important role providers should play in weight-loss counseling for their patients to promote overall health.

### Limitations

Due to its retrospective design, this study may inherit bias from loss to follow-up and PROM survey noncompliance. It is possible that bias towards a White, female predominance may limit the generalizability of our results, and our PROM scores may not completely capture male or non-White patients. Additionally, though PROM score collection occurred for all patients during clinic visits within the study period, PROM survey administration at standardized time intervals was not able to be conducted, introducing variability in follow-up time among patients. Further, because all patients did not follow a regular follow-up schedule, we were unable to analyze postoperative PROM scores over multiple, longer-term timepoints, and may not have completely captured the full extent of score improvement. Importantly however, the aim of the current study was to compare relative overall improvements across BMI classes, rather than absolute improvements at a specific time point, so that the general association between obesity and PROM score changes could be assessed. Lastly, our analyses only described subjective outcomes in the form of PROMs, which may differ from objective clinical outcome differences across BMI groups.

## Conclusion

Obese patients undergoing TKA report worse preoperative and postoperative scores for most measures of functional status and health, despite achieving comparable pre/post-Δ improvements in PROMIS measures of pain, mobility, function, and mental health with surgery. Elevated BMI is independently associated with greater pre/post-Δ improvements in KOOS, JR and PROMIS physical health scores compared to normal weight patients. Obese patients may achieve superior benefit from TKA as compared to non-obese patients. Institutions considering limiting patient eligibility for TKA based on BMI should include patient-reported improvements for obese and severely obese patients into their risk–benefit analysis.

## Data Availability

The datasets generated during and/or analysed during the current study are not publicly available due to patient confidentiality but select, blinded data can be made available from the corresponding author on reasonable request.
